# Proximal Phalanx Enchondroma in a Pediatric Patient: Curettage, Liquid Nitrogen Cryotherapy, Iliac Crest Autograft, and Microfragment Plate Fixation

**DOI:** 10.7759/cureus.114768

**Published:** 2026-08-18

**Authors:** Mario R López Monzón, Arnold B Palma, Rogelio A Morataya, Luis R Calvo

**Affiliations:** 1 Department of Orthopedics and Traumatology, Hospital General San Juan de Dios, Ciudad de Guatemala, GTM

**Keywords:** benign bone tumor, benign hand tumor, bone graft, cryotherapy, enchondroma, liquid nitrogen, proximal phalanx

## Abstract

Enchondromas are benign, slow-growing cartilaginous tumors, with the phalanges of the hand being the most common site. Although standard surgical treatment consists of curettage and bone grafting, the combined use of liquid nitrogen cryotherapy and internal fixation in pediatric patients with extensive cortical involvement remains poorly documented in the literature. A 14-year-old male patient presented with progressive deformity of the middle finger of the right hand over a period of more than one year. Radiographs demonstrated an expansive lytic lesion in the proximal phalanx measuring 21.85×13.00 mm, with marked expansion and thinning of the ulnar cortex. Surgical treatment consisted of a dorsal approach, thorough manual and high-speed burr curettage, liquid nitrogen cryotherapy in two freeze-thaw cycles, filling of the defect with iliac crest cancellous autograft, microfragment plate fixation, and repair of the ulnar collateral ligament. Histopathological examination confirmed the diagnosis of enchondroma. At six months of follow-up, the patient was pain-free and had achieved complete range of motion (total active motion of 255°), preserved ligamentous stability, and radiographic evidence of graft integration, without cryotherapy-related complications. In this case of proximal phalanx enchondroma with significant cortical involvement in a pediatric patient, the combination of extensive curettage, liquid nitrogen cryotherapy, iliac crest autograft, and microfragment plate fixation proved to be a safe and effective surgical strategy. Extended follow-up is required to evaluate the risk of local recurrence.

## Introduction

Enchondromas are benign cartilaginous neoplasms that arise from remnants of the growth plate cartilage trapped within the medullary cavity. They account for approximately 10%-25% of all benign bone tumors and represent the most common primary bone tumor of the hand [1,2]. The proximal phalanx is the most frequent site, followed by the metacarpals and middle phalanges, with a predilection for the index and middle fingers [3].

Clinically, most cases involving short tubular bones are discovered incidentally or present with progressive deformity or pathological fracture. Radiographically, they appear as well-defined expansile lytic lesions with punctate chondroid calcifications, cortical thinning and expansion, and absence of periosteal reaction [4].

The standard surgical treatment is intralesional curettage with or without bone grafting. However, reported recurrence rates range from 2% to 15%, prompting the use of adjuvant therapies such as high-speed burring, phenol instillation, and liquid nitrogen cryotherapy to eradicate residual tumor cells from the cavity walls [5,6].

Although liquid nitrogen cryotherapy has been described primarily as an adjuvant in long bones, its use in the proximal phalanx of a pediatric patient - combined with iliac crest autograft reconstruction and microfragment plate fixation - remains a rarely reported technical strategy in the literature. The purpose of this case report is to describe the surgical technique employed and the functional and radiological outcomes achieved.

## Case presentation

A 14-year-old male patient with no relevant medical history was referred to the Department of Orthopedics and Traumatology at Hospital General San Juan de Dios, Guatemala City, with a one-year history of progressive deformity of the middle finger of the right hand and no antecedent of trauma. Physical examination revealed fusiform swelling over the proximal phalanx, without erythema or signs of acute fracture, and with preserved joint mobility.

**Figure 1:**
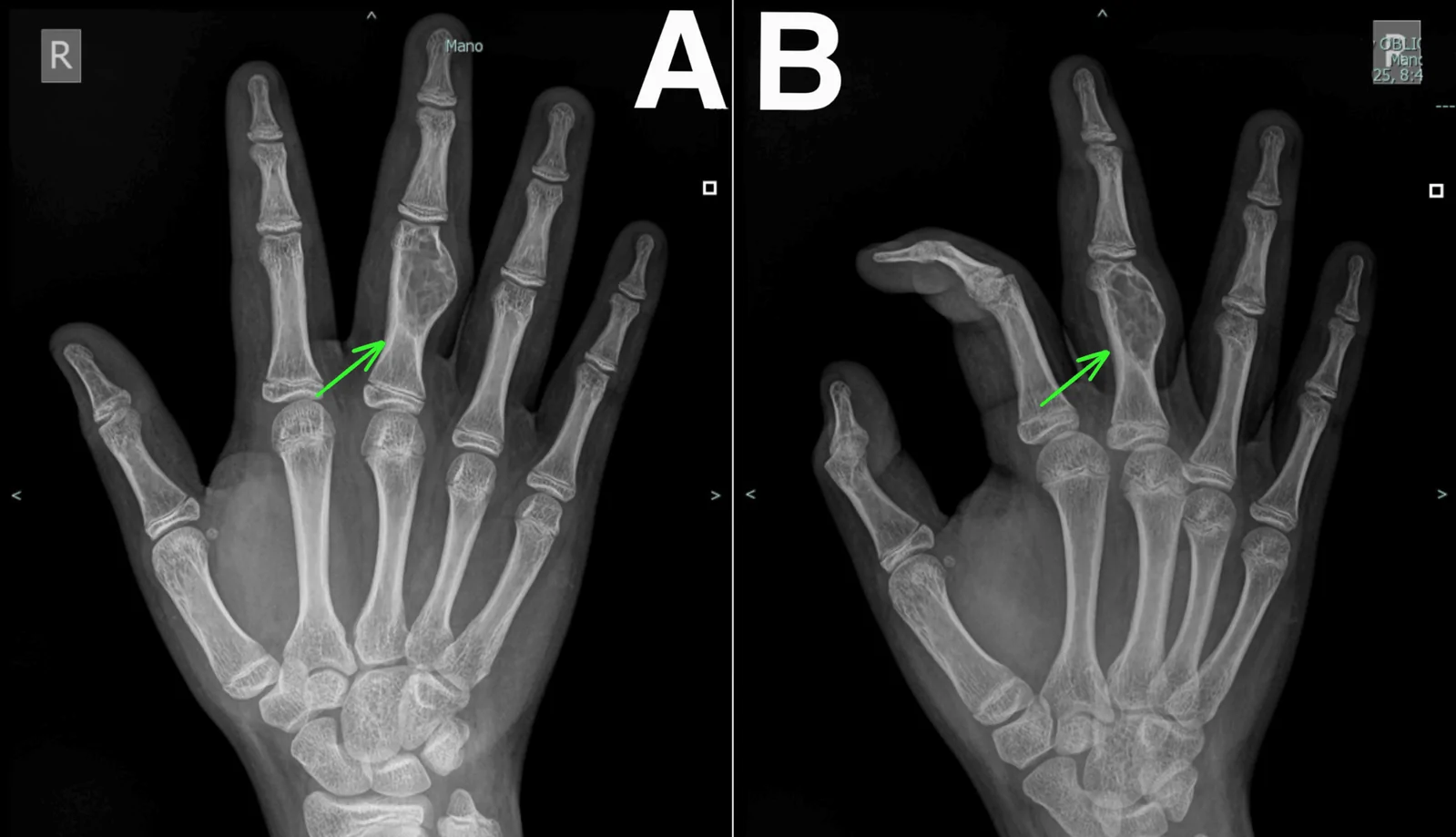
Preoperative radiographs. (A) Anteroposterior view. (B) Oblique view. Expansile lytic lesion in the proximal phalanx of the middle finger with expansion of the ulnar cortex and internal chondroid calcifications.

Plain radiographs in anteroposterior and oblique projections demonstrated a well-defined expansile lytic lesion measuring 21.85×13.00 mm in the proximal phalanx of the middle finger, with internal chondroid calcifications and marked thinning and expansion of the ulnar cortex (Figure 1). No pathological fracture or periosteal reaction was noted. Magnetic resonance imaging and computed tomography were not performed, given the characteristic radiographic appearance.

Written informed consent was obtained from the patient’s legal guardian for the surgical procedure and for the publication of clinical and imaging data.

Elective surgery was performed under regional and general anesthesia. The patient was positioned supine with the upper limb extended on a hand table and a pneumatic tourniquet applied to the forearm. A dorsal approach was made over the proximal phalanx of the middle finger. After incising the skin and subcutaneous tissue, the extensor apparatus was identified and preserved by being retracted. A dorsal cortical window was created, followed by thorough manual curettage using curettes of varying sizes.

High-speed burr debridement was then performed to remove residual cartilaginous tissue from the cortical walls. Intraoperative exploration revealed critical thinning and complete loss of structural integrity of the ulnar cortex secondary to tumor expansion, resulting in marked segmental instability; the compromised cortex was therefore resected. Liquid nitrogen cryotherapy was applied using swabs in two freeze-thaw cycles, with the protection of adjacent soft tissues using warm saline-soaked gauze. The cavity was thoroughly irrigated with saline solution.

The resulting bone defect, enlarged by resection of the incompetent ulnar cortex, was filled with cancellous autograft harvested from the ipsilateral iliac crest and progressively impacted. Given the extent of the cortical defect and residual instability, osteosynthesis was performed using a low-profile microfragment plate with bicortical fixation to ensure mechanical stability and protect the graft during incorporation. The ulnar collateral ligament was repaired with non-absorbable suture to restore joint stability. Wound closure was performed in layers.

**Figure 2:**
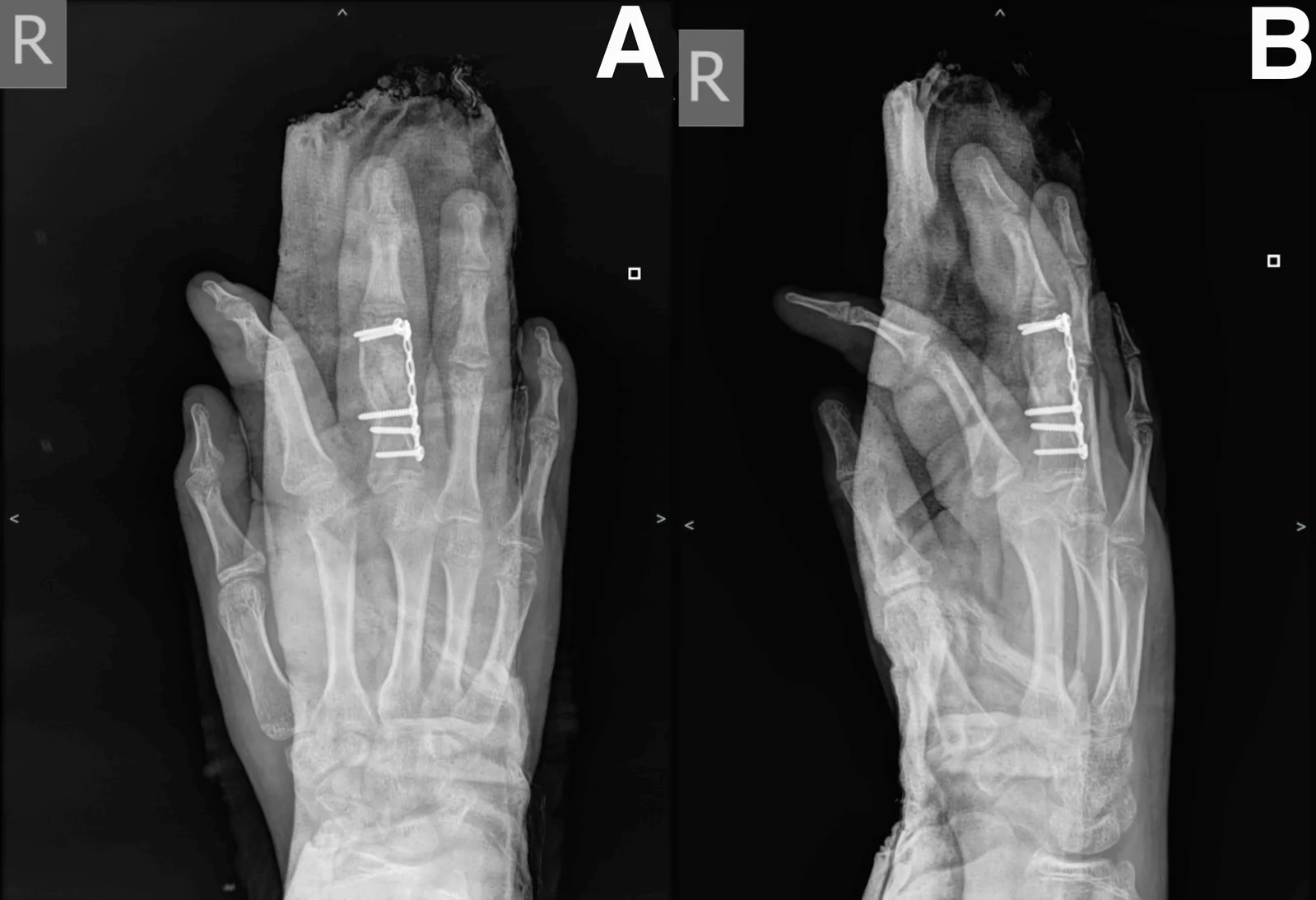
Immediate postoperative radiographs: (A) anteroposterior view; (B) oblique view.

The tumor specimen was sent for histopathological analysis, which confirmed the diagnosis of enchondroma. The postoperative course was uneventful. Supervised active and passive mobilization was initiated at the second postoperative week. At the six-month follow-up, the patient was asymptomatic, with no pain at rest or during activity. The range of motion assessment revealed proximal interphalangeal joint flexion of approximately 100° with full extension, metacarpophalangeal joint flexion of 85°, and distal interphalangeal joint flexion of 70°, resulting in a total active motion (TAM) of 255° (Figure 2).

**Figure 3:**
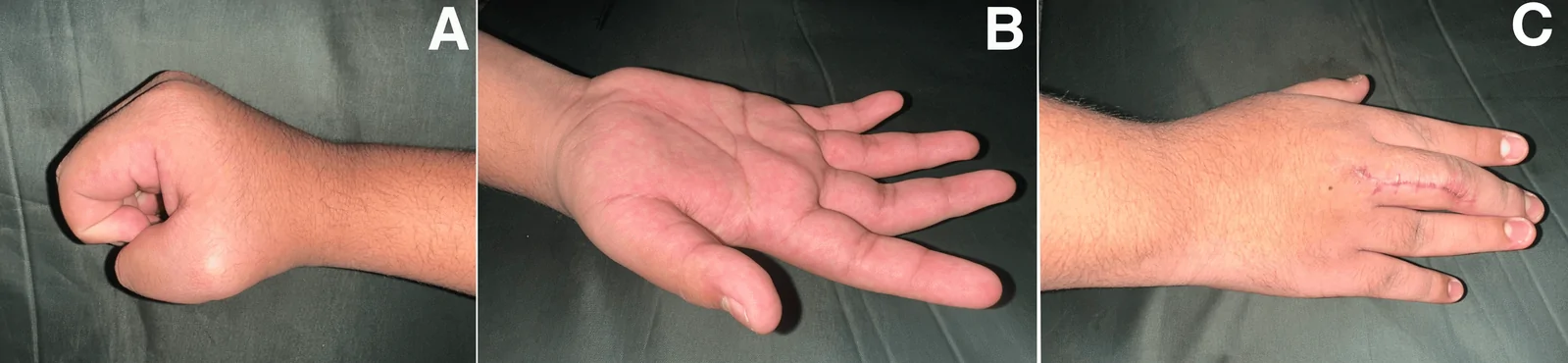
Clinical photographs of finger mobility at six-month follow-up: (A) complete active flexion; (B) complete active extension; (C) postoperative scar.

**Figure 4:**
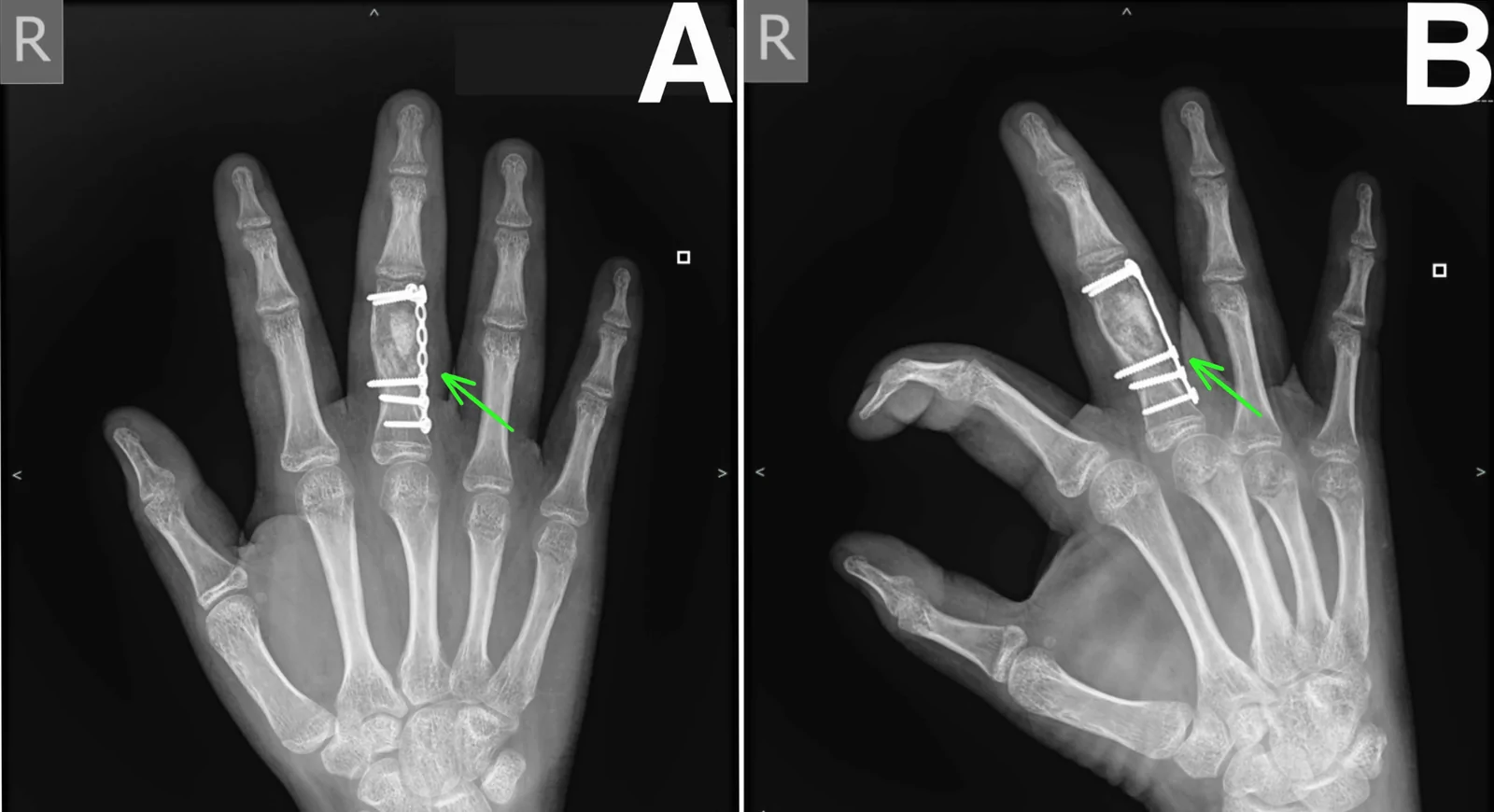
Six-month follow-up radiographs: (A) anteroposterior view; (B) oblique view. Progressive incorporation of the iliac crest autograft with ossification of the bone defect and microfragment plate in situ.

Ligamentous stability was preserved, and the patient had resumed unrestricted school activities. Follow-up radiographs demonstrated adequate graft incorporation and remodeling, with progressive ossification of the defect, cortical consolidation, and restoration of the trabecular architecture of the phalanx (Figures 3, 4). No cryotherapy-related complications, including pathological fracture, late osteonecrosis, or soft-tissue injury, were observed.

## Discussion

Enchondroma of the proximal phalanx is the most common benign bone tumor of the hand. Although its peak incidence occurs in young adults, it can manifest in adolescence with progressive deformity, as observed in the present case. Intraoperatively, critical compromise of the ulnar cortex with complete loss of structural integrity was identified, necessitating modification of the initial reconstructive strategy and underscoring the importance of surgical preparedness to adapt the procedure according to intraoperative findings.

In lesions with extensive cortical involvement, such as this case (involving approximately 70% of the bone volume), simple curettage alone may be insufficient because of the risk of instability and postoperative fracture. Table 1 summarizes the principal surgical options and justifies the selection of a more aggressive technique in this patient.

**Table 1:** Comparison of surgical techniques for proximal phalanx enchondroma. Source: Authors' own elaboration based on reviewed literature [5-10].

Technique	Advantages	Disadvantages	Fixation	Recurrence
Simple curettage	Simple; no donor site morbidity	Instability in large cavities	Not required	2-15%
Curettage+bone cement	Immediate stability; low cost	No biological properties	Optional	~5%
Curettage+synthetic substitute	No donor site morbidity	Inferior osteogenic properties	Optional	~5%
Curettage+allograft	Biological properties; no donor site	Limited availability in low-resource settings	Optional	~5%
Curettage+iliac crest autograft	Optimal osteogenic, osteoinductive and osteoconductive properties	Donor site morbidity	Recommended in large defects	~2-5%
Present case (curettage+adjuvants+autograft+plate)	Maximum tumor destruction; stability; early mobilization	Greater surgical complexity; donor site morbidity	Microfragment plate	Under follow-up

Liquid nitrogen cryotherapy, delivered in two freeze-thaw cycles, was employed to maximize the eradication of residual tumor cells within the cavity walls. Although this adjuvant technique is better documented in long bones, its application in pediatric phalanges remains uncommon [6]. No cryotherapy-related complications were observed in this case, including pathological fracture, delayed osteonecrosis, or soft-tissue injury. This favorable outcome is likely attributable to meticulous protection of the perilesional soft tissues with warm saline-soaked gauze and the relatively limited size of the phalanx, which restricted the area of cryogenic exposure.

## Conclusions

In this pediatric patient with a proximal phalanx enchondroma and extensive cortical involvement, thorough manual and high-speed burr curettage, followed by liquid nitrogen cryotherapy in two freeze-thaw cycles, iliac crest cancellous autograft reconstruction, and low-profile microfragment plate fixation resulted in an uneventful postoperative course and complete functional recovery at short-term follow-up, achieving a total active motion (TAM) of 255° at six months. Intraoperatively, limited resection of the thinned ulnar cortex was performed to achieve complete tumor clearance. This finding necessitated primary repair of the ulnar collateral ligament and influenced the final fixation strategy, highlighting the importance of intraoperative adaptability. Medium- to long-term follow-up remains essential to confirm the absence of local recurrence.
